# What can global health institutions do to help strengthen health systems in low income countries?

**DOI:** 10.1186/1478-4505-8-22

**Published:** 2010-06-29

**Authors:** Dina Balabanova, Martin McKee, Anne Mills, Gill Walt, Andy Haines

**Affiliations:** 1 London School of Hygiene and Tropical Medicine, 15-17 Tavistock Place, London WC1H 9SY, UK

## Abstract

Weaknesses in health systems contribute to a failure to improve health outcomes in developing countries, despite increased official development assistance. Changes in the demands on health systems, as well as their scope to respond, mean that the situation is likely to become more problematic in the future. Diverse global initiatives seek to strengthen health systems, but progress will require better coordination between them, use of strategies based on the best available evidence obtained especially from evaluation of large scale programs, and improved global aid architecture that supports these processes. This paper sets out the case for global leadership to support health systems investments and help ensure the synergies between vertical and horizontal programs that are essential for effective functioning of health systems. At national level, it is essential to increase capacity to manage and deliver services, situate interventions firmly within national strategies, ensure effective implementation, and co-ordinate external support with local resources. Health systems performance should be monitored, with clear lines of accountability, and reforms should build on evidence of what works in what circumstances.

## Introduction

Global Health Initiatives, such as the Global Fund to fight AIDS, Tuberculosis and Malaria (GFATM), the Global Alliance for Vaccines and Immunization (GAVI) have raised large sums of money for essential health care and have challenged previous notions of what is possible. However the ability of countries to spend the new funds effectively is often constrained by fundamental weaknesses in their health systems [1-3]. Hence, commentators have questioned the fragmentation and competing priorities of many disease-specific programs [4] and whether they succeed in reaching the poorest groups [5], while there are broader concerns about their ability to take account of national contexts [6]. There is a clear need to strengthen health systems, given their core role in delivering services and, especially, their ability to close the knowledge-action gap [7]. Yet, much of what is written has concentrated on diagnosis, with rather less attention to solutions [2,8]. An extensive review published in 2009 highlighted the limited evidence that Global Health Initiatives had succeeded in improving national health systems [9].

Here we review the challenges that face those seeking to strengthen health systems. Drawing on the framework proposed in the 2000 World Health Report [10], we view strengthening as being directed towards the ability of the entire system to collect, pool, and spend the necessary finances to become sustainable and equitable, to deliver effective, appropriate, and equitable care, to generate the necessary resources (such as a trained workforce) to make this happen, and to provide the stewardship to ensure its effective governance. We distinguish this from the partial strengthening that has so far been undertaken by several agencies involving directed support for their own activities, support for a limited set of health systems functions necessary for the delivery of their own activities, or integration of their activities into the existing health system [11].

A major conclusion is the urgent need for evaluative research to discover what works and in what circumstances. First, however, we need to understand what the problems are. We therefore examine the main health system functions according to the categoriation set out above, describing key issues that emerge.

The paper combines a review of literature identified from an initial search of PubMed using combinations of the terms "health system", strengthen*, full and abbreviated forms of GFATM and GAVI, vertical and horizontal, with follow up of relevant citations, and from the literature collections amassed by the authors over many years of working in this area (which include a substantial number of official publications from agencies). The material used is limited to that published in English. The review does not seek to provide an exhaustive catalogue of everything that has been written on this topic, but it does capture the essential elements and provides a range of examples to illustrate them.

### Health system diagnosis: what are the key problems?

#### Financing

Those in most need of health care are often least able to afford it or to judge the quality of care they obtain. Financing of health systems has three main elements: collection of funds, pooling them, and purchasing effective care. All three elements often fail. In poor countries weak infrastructure and enforcement systems mean that payment of taxes and other contributions are essentially optional, while in others, what capacity that exists to collect taxes is not fully utilized. Most care is paid for by a combination of external funds, typically earmarked for specific conditions, and private out of pocket payments. Global Health Initiatives have brought substantial additional resources for particular activities [12]. However, this means that numerous donor funds and agencies fund different elements of health systems, with poor co-ordination impacting adversely on national systems, fragmentation of resources and high transaction costs [1]. This is often worst in the poorest countries [13]. In addition, there is now growing evidence that international aid flows displace overall domestic health expenditure [14,15], especially in countries in receipt of loans from the International Monetary Fund[16].

Failure of co-ordination increases burdens on already pressured national institutions [17]; thus when donor projects increased from a few hundred to several thousand in Tanzania in the late 1990s the government had to prepare 2,400 reports every quarter, with senior officials hosting 1,000 meetings annually[18]. Yet the overall level of aid remained almost unchanged. The resulting strain on the system hampered the ability to manage and led to the introduction of an annual four-month "mission holiday" free from meetings with donors.

International funding channels, whether through vertical mechanisms or general budget support, are often disconnected from local revenue-generating initiatives, such as community based health insurance. Scarce local funds inadequately fill gaps that external funders decline to support. Multiple financing arrangements make resource pooling impossible and the myriad of individual transactions from out of pocket payments precludes strategic purchasing of services from providers. A second problem is the plight of families faced with catastrophic expenditure due to serious illness [19]. Both have profound consequences for the state; first because it is difficult to tackle inefficient use of scarce resources and second because of the macroeconomic consequences as families hoard money rather than reinvesting it in economic development [20].

#### Provision

Traditional vertical programs can succeed in scaling up basic interventions such as vaccination as long as the external funding lasts, but they have failed to respond to the complex challenges now confronting them. Conditions such as AIDS, diabetes, and schizophrenia require long term relationships between patients and multi-disciplinary teams, access to different levels of care, and reliable supplies of pharmaceuticals [21]. Those seeking to scale up treatment for AIDS have recognized that funding for drugs is not enough [22], a lesson already learned by those trying to ensure treatment for insulin dependent diabetes [23]. There are strong theoretical arguments favoring integrated management of a range of chronic diseases in primary care settings [24], and although the evidence for integrating targeted programs with mainstream services is limited [25], there examples of how positive synergies can be achieved. Thus, investment in services for HIV/AIDS in Haiti [26] and Rwanda [27] improved access to antenatal services and family planning, as the latter benefitted from investment in new facilities, laboratories, and training of health workers. In the African Programme for Onchocerciasis Control, locations that added other health interventions to their activities achieved higher uptake of the drug Ivermectin than those that did not [28].

#### Resource generation and priority setting

Money is important but not sufficient for strengthening health systems. Many health systems have limited absorptive capacity. They face shortages of key staff, migration, and low skill levels [29]. Human resource policies must include initial training and life-long learning, skill-mix and career progression, with appropriate incentives and working conditions [30]. However, these policies must take account of the substantial obstacles to change imposed by obsolete structures, institutions, and beliefs [30,31]. Weak procurement and distribution systems adversely affect drug supply, exacerbated by counterfeiting and corruption. Government taxes, inefficient procurement, mark-ups along the distribution chain, and, in some middle income countries, fee-splitting between pharmacists and physicians, inflate drug prices [32]. An absence of capital planning and health technology assessment means that technology is often purchased for its ability to generate fees rather than its appropriateness. Public health capacity and information systems are weak.

Necessary resources go beyond people and things. Health systems should also have systems to generate the knowledge resources they need to function optimally. Governments attending the 2008 Global Ministerial Forum on Research for Health in Bamako, Mali, agreed on the importance of building research infrastructure into health systems [33,34]. It is only with these knowledge resources, which encompass the production of research and the creation of systems to ensure that it is used, that health systems can deliver effective care that is appropriate for the context in which it is being delivered.

#### Stewardship

The stewardship function [35], has been especially weak. In many countries failures of stewardship in the health sector reflect wider failures of governance in the country as a whole. The situation is worst in fragile states and those where democracy is weak, and is exacerbated by the existence of parallel sub-systems, especially where these are essentially independent of the state [36]. In many countries health care is seen as among the most corrupt sectors, manifest in a myriad of ways from overpriced procurement of supplies to informal payments and sales of counterfeit medications. The net effect is to increase even further the mismatch between need and supply [37]. Unfortunately, many health systems are unable to establish the systems that would be needed to prioritize, plan and deliver the effective policies.

#### The wider world

Many health systems are already unable to respond adequately to contemporary challenges yet emerging pressures will intensify. Populations are changing, through both ageing and migration, with the latter likely to be driven partly by environmental degradation. The threat from newly and re-emerging infections is ever present. Products underlying the epidemic of chronic disease in the west, such as tobacco and energy dense processed food, are being marketed aggressively in the developing world. Growing road traffic is resulting in increasing injuries and sedentary lifestyles.

The success of existing initiatives tackling major killers such as common childhood illnesses means that the burden of disease is now dominated by chronic disorders requiring complex packages of care. The development of integrated models of care for people with complex chronic conditions has challenged the richest countries [38]; there is much less experience in developing countries [39]. Investment has often been culturally inappropriate and useful innovations have been difficult to implement [40].

### Current efforts

The response to contemporary global challenges has led to proliferation of new institutions and calls for a new 'global architecture' [41]. Several attempts have been made to improve aid effectiveness, by raising more funds, harmonizing efforts between donors, and aligning aid with national priorities. The Paris Declaration, the Accra Agenda for Action, UNAIDS' 'Three Ones', and the International Health Partnership all sought to bring a greater sense of coherence to both global and national policies. However, translating goals of harmonization and alignment into practice at country level has been slow and difficult, reflecting competing or conflicting interests and power of different partners. Put simply, some donors are more powerful than others, either as a consequence of the resources they command or because of their status in the geo-political context. For example, a development agency from a particular country will be more influential if that country is also contributing to the defence of the recipient country against internal or external security threats. Poor coordination of efforts by external partners continues to impose high transaction costs on national health systems [42,43] although there are increasing examples where Global Health Initiatives support national co-ordination [44].

Donor alignment and coordination and strengthening of health systems are being pursued through several global initiatives (Table 1). The International Health Partnership, launched in September 2007, brought together major donors (the Health 8), western governments, and health ministers in recipient countries, to examine how to expand coverage of essential interventions and improve health outcomes, drawing on the strengths of public, private and voluntary sectors. It also provided the secretariat for the recent Taskforce on Innovative International Financing for Health Systems [45].

**Table 1:** Examples of the over 75 global health partnerships and initiatives attempting to improve coordination of effort among donors and between donors and countries

Global/regional level		
*Signed Agreements*	Paris Declaration on Aid Effectiveness (2005)	Harmonization of donors and alignment with national priorities Indicators for Monitoring & Evaluation

	International Health Partnership Global Compact (2007)	Improving coordination on national health plans

	The 'Three Ones'	Harmonization and alignment in HIV/AIDS. UNAIDS, the Global Fund, and other agencies. • one agreed HIV/AIDS action framework which provides the basis for coordinating the work of all partners; • one national HIV/AIDS coordinating authority with a broad-based multi-sectoral mandate; • one agreed country-level system for monitoring and evaluation.

	Global Task Team on Improving AIDS Coordination among Multilateral Institutions and International Donors (2005)	

	Global Implementation Support Team (2007)	

*Processes*	Health 8 agencies (H8)	Gates Foundation, GFATM, GAVI Alliance, WB, WHO, UNAIDS, UN Population Fund, UNICEF have 6 monthly informal meetings to discuss coordination and aid effectiveness issues. Agreed in July 2007 to a coordinated health systems strengthening effort, playing a central role in co-ordination of IHP+.

	Global Campaign for the Health MDGs (26 September 2007)	■ The International Health Partnership ■ The Catalytic Initiative (November 2007), by Canada and UNICEF ■ Results-Based Financing Initiative (November 2007), by Norway and the World Bank. ■ Providing for Health Initiative on social health protection, 2008, by Germany and France ■ Global Leaders Network ■ Deliver Now for Women and Children

	International Health Partnership and Related Initiatives (IHP+) (2007)	Interagency Core Team (based in WHO & WB: and the Harmonisation for Health in Africa, based in WHO's Africa Regional Office) Scaling-up Reference Group - SURG (representatives of the H-8 agencies, civil society and development partners)

	Global Health Workforce Alliance	

	Health Metrics Network	

	Partnership for Maternal, Newborn and Child Health	

	Medicines Transparency Alliance (MeTA)	

	Disease specific coordination mechanisms	e.g. Stop TB Alliance, and Roll Back Malaria

**National level**	SWAps	

	PRSps	(health system interventions integrated in a poverty reduction strategies)

	IHP+ country compacts	

	Country Coordinating Mechanisms for GF	

	One-UN, 2007	(all UN agencies under one roof, a lead agency, one budgetary framework)

	UN 'cluster approach'	In emergency settings/chronic conflict whereby one UN agency is responsible for taking the lead in coordination with the government.

This commitment is also apparent in the funding strategies of major donors, including the GFATM and the GAVI. Indeed the Taskforce process stimulated the Global Fund, GAVI Alliance and World Bank to propose a joint mechanism for investment in health systems. Yet applications to the GFATM focused primarily on health system strengthening have so far had limited success [46]. Their guidance on what might be supported may not be aligned with the priorities of recipient countries and thus may fail to secure political commitment. The World Bank Strategy for Health, Nutrition, and Population Results emphasizes "the need to ensure synergy between enhanced priority-disease financing and strengthening of health systems, essential for achieving results and improving DAH [Development Assistance for Health] effectiveness on the ground"[47], although critics have argued that the Bank's policies on issues such as the public-private mix expose fundamental contradictions with these aims [48]. The United Kingdom's Department for International Development has placed health system strengthening at the heart of its health strategy, seeing health systems as key to channeling investment to the poor [49]. Effective and accessible health systems are viewed as a key step towards long-term objectives such as poverty alleviation.

These approaches are being accompanied by a greater emphasis on the need to develop sustainable financing, including well-functioning collection systems and risk-pooling arrangements [50]. The role of donor support more fundamentally in supporting national health financing systems has also been advocated, including the scope for increasing demand through vouchers or payment of insurance premiums. This approach is also receiving support from country-level plans and poverty strategies within SWAps (Sector wide approaches) and Poverty Reduction Strategy Papers (PRSP). Poverty Reduction Strategy Papers (PRSPs) are documents required by the International Monetary Fund and World Bank before a country can be considered for support within the Highly Indebted Poor Country programme, designed to relive the debt burden of the poorest countries. They are prepared by countries by means of a participatory process involving domestic stakeholders as well as external development partners, including the World Bank and International Monetary Fund.

The Sector-Wide Approach (SWAp) is a mechanism whereby funds from different projects contribute to a sector-specific (such as health or education) umbrella that is tied to a defined sector policy under a government authority. It calls for a new form of partnership between governments and development agencies, in which the leadership role of the former is strengthened. Key characteristics include clear ownership and leadership of the programme by the national government and a common effort by external partners to support that programme, including provision of all or a major share of funding for the sector, in support of the government's unified policy and expenditure programme.

The high level Taskforce on Innovative International Financing for Health Systems has called for an additional $10 bn per year of external assistance by 2015, as well as substantially increased domestic public financing for health [45].

### What needs to happen within countries to strengthen health systems?

Many of the actions required flow directly from weaknesses identified earlier. The health systems framework set out in the 2000 World Health Report draws attention to the need to focus on otherwise neglected areas, such as mobilizing resources, risk pooling, and stewardship. In some areas the degree of underinvestment is clearly acknowledged, for example the need to increase numbers of health workers and appropriate 'task shifting'[29]. It is, however, necessary to look at the system as a whole, avoiding simplistic checklists for reform [51]. This demands a coherent vision at the country level of the way forward, which can then be operationalized through integration of programs for health problems that share common characteristics, such as chronic diseases, interlinkage of inputs, and coordination between levels of care, sectors, and policies, all based on effective communication between patients (and communities), practitioners, and policy-makers. Strong country leadership and political commitment have been crucial factors in integrating disease-specific programs within broader delivery systems (e.g. HIV scale-up in Brazil and Thailand), within national primary health care strategies and information systems (Dominica), and in creating linkages to training and optimizing skill mix of workers (Ethiopia, Malawi)[52].

The traditional distinction between vertical (disease-specific) and horizontal approaches is being reassessed. Each has strengths and weaknesses. Where health systems are extremely weak, vertical programs can play an important role, but they need to be designed in ways that act as a catalyst for wider system change (as is being attempted by the GFATM and GAVI), becoming interwoven in a matrix with horizontal programs [2]. It is also possible to sequence vertical and horizontal approaches in ways that enhance outcomes and build sustainable and locally relevant long term capacity [8]. One manifestation are the so-called 'diagonal programs'[53] being explored by the GFATM, where system-wide investment includes a degree of support to specialized disease functions [54]. These involve a "strategy in which we use explicit intervention priorities to drive the required improvements into the health system, dealing with such generic issues as human resource development, financing, facility planning, drug supply, rational prescription, and quality assurance." [55]

Taking the example of the Global Fund, Ooms et al. argue that diagonal approaches to financing (aiming for disease-specific results through improved health systems) can be an intermediary step towards horizontal financing involving coordination and integration of disease-specific interventions into the broader health systems [14].

According to WHO, diagonal approaches reconcile the need to keep some specialised functions while recognising that programmes and their scaling up require stronger health systems. A related concept is that of "integration of service delivery", links between prevention and treatment, between different stakeholders, between public, voluntary and private sectors, and between levels of the health system.

Such approaches offer scope to benefit from synergies in the management of different diseases, particularly for complex chronic disorders such as AIDS, diabetes, and cardiovascular disease. All require functioning pharmaceutical distribution systems and mechanisms to train staff in areas as diverse as management, quality assurance, information systems and palliative care but it is wasteful to organize each of them separately. Yet achieving synergies will often require considerable effort to overcome historical patterns of provision, based on established funding streams, hierarchies, training and clinical routines that serve the interests of the provider rather than the patient.

This highlights the importance of paying attention not only to the design of health systems strategies but also to the implementation of change and the ways of overcoming the factors that hamper it [56]. There is a need to acknowledge the diversity of skills, motivations, and beliefs among those with a stake in health systems, understanding how community-level influences, professional interests and informal relationships between actors influence how programs are implemented and the degree to which they achieve their goals.

For this to happen, there is a need to realign the focus of much existing research, based on a better understanding of how health systems develop and change in different contexts. Too often in the past it has been assumed that a policy developed in one setting will work in another and, even when it is clear that the policy has failed, there is little interest in asking what it was about the context into which it was transplanted that caused it to do so. This requires a greater understanding of the concept of path dependency whereby, in the absence of a severe shock to the broader political system, the development of a health system is constrained by the history, culture, economic development, and institutional structures (especially labor relations, systems of government, and the rule of law) of the country in which it is situated [6,57]. These must be understood if realistic reform strategies are to be developed. Hence, research on barriers to implementation must consider not only the outcomes and impacts of reform outcomes but also the processes involved and the context in which they take place.

### What changes are needed in the global aid architecture and how can they be implemented?

Improved health systems functioning demands change in the global aid architecture. Key issues include coordination, comprehensiveness, continuity, capacity, and accountability.

Health systems strengthening can give the impression of becoming simply the latest fashion, with many actors now becoming engaged through funding, agenda setting, provision of technical advice, and oversight of implementation. This can bring benefits, as each actor can have competitive advantages, based on skills, areas of expertise, and established links but also poses challenges.

These actors must fulfill a number of different roles. While almost all provide some degree of technical assistance, some are primarily donors providing funds to strengthen health systems, some of whom will focus on specific diseases or interventions, such as the GFATM, GAVI, and PEPFAR, while others take a broader perspective on the overall health system (and beyond), typified by bilateral development agencies such as DFID, DANIDA, and SIDA. Both types have a responsibility to ensure that they recognize the complex interlinkages between their various activities. They also must ensure that the evidence base for policy and practice is strengthened, available evidence is acted upon in programs that they fund, and that these programs contribute to health system strengthening in effective and transparent ways. Examples include developing a cadre of health workers that can contribute to health system priorities and not just a single disease, supporting sustainable health system financing mechanisms, and putting in place quality assurance systems which can cover a range of health outcomes. However, efforts by donors to invest in disease-specific programmes that also contribute to system-building are hampered by the limited evidence on what activities actually result in strengthening health systems and under what conditions [11].

Others have a normative role in addition to the technical assistance they provide at country level. These include the WHO and, in some respects, the World Bank. Their role should be to ensure that the best available evidence is used to inform the development and implementation of effective policies, activities that have recently been endorsed by the leaders of eight of the global health agencies, including the WHO, World Bank, GFATM, and GAVI [58].

These roles are clearly complementary but the increased global engagement in health systems strengthening is leading to considerable fragmentation in priorities and strategies [47,59]. The challenge is how to reap the benefits of this diversity while ensuring that activities are, at best, aligned with each other and with what countries want (i.e. follow country priorities and needs and are context specific) and, at least, are not in conflict. There is certainly a view among many recipient countries that this process must start at country level and build on existing coordination processes nationally [60].

This analysis implies a need for leadership. Yet it is far from clear where this leadership should come from. Although the WHO views itself, and is viewed by many others, as occupying a position of leadership in global health, the complementarities of the many specialized institutions involved means that there may be different leaders in different situations. Rather, it seems that there is a need for dynamic partnerships in which different institutions lead in different areas, but in ways that support rather than undermine the work of others. These partnerships should support leadership by developing countries and South-South coalitions as far as possible [60]. Where there is strong country leadership, as in Rwanda, partnerships do work well. Furthermore, involvement of the international private and voluntary sectors in these coordination processes is vital given their important role in the health systems of many countries.

There are already some positive developments seeking to improve the effectiveness and predictability of aid flows and reduce duplication across donors and implementing agencies [61]. However, the linkages between initiatives are not always clear. Coordination is also needed within organizations: there is a risk that health systems support is viewed as yet another initiative, separate from what is taking place to provide disease-specific support. There is encouraging evidence that this is being avoided, for example in the Global Health Initiatives [62], health systems support is beginning to be viewed as cutting across programs within agencies (GFATM, GAVI) rather than as free-standing units within agencies, although experience with such arrangements is so far limited.

The diversity of actors in part reflects a multiplicity of interests. Agencies such as the GFATM and GAVI have clear mandates to focus on specific elements of health care. Non-governmental and private donors have also carved out niches, often focused on individual diseases. However, policies on health systems are more vulnerable than disease control policies to ideological influences, as evident in controversies over user fees and social health insurance, with donors often influenced by their own health service traditions and cultures. Effective coordination requires agreement on both investment priorities for health systems strengthening and implementation strategies. Aligning disbursement procedures is likely to pose challenges and will require significant commitment from both donor and recipient governments and a willingness to sacrifice some direct control over funding for greater aggregate impact. This will require greater independent evaluation of aid effectiveness over sustained periods.

Experience with SWAps highlights both the importance and difficulties of pooling resources, agreeing priorities, coordinating reporting systems, monitoring, and embedding aid flows within national plans and budgets as a pre-requisite for creation of comprehensive approaches. Although experience has varied, where SWAps have been effective, as in Uganda, they have helped governments to develop strategic oversight over policies and resources, while working in partnership with donors and implementing agencies [63]. Yet there is a constant threat from disease-led funding focused on short project cycles, with "deliverables" that are not aligned with national priorities [64] but which serve donor interests to demonstrate quick results. An analysis of Overseas Development Assistance for maternal, neonatal and child health demonstrates that there was virtually no change between 2003 and 2006 in the share of project funding, and that project funding dominates sector and budget support [65] (Table 2). Experience with SWAps also demonstrates the technical challenges; differences in accountability and reporting mechanisms have prevented Global Health Initiatives being placed within them [62].

**Table 2:** Official Development Assistance (ODA) to maternal, newborn and child health by aid modality 2003-2006 in constant 2005 US$

Aid Modality/Purpose of Project	2003		2004		2005		2006	
	
	ODA	%	ODA	%	ODA	%	ODA	%
General budget support	51,044	2%	86,216	4%	50,358	2%	68,650	2%

Health sector support	31,036	1%	44,079	2%	66,722	2%	123,060	3%

Projects	2,037,302	96%	1,926,579	94%	2,818,348	96%	3,289,993	95%

Note: For the 68 recipient countries identified by the Countdown group as a priority in terms of child mortality and maternal mortality. ODA: Overseas Development Assistance Source: [65]

Some have argued for an explicit focus on the extent to which funding achieves demonstrable synergies [22], with others linking this to calls for improved monitoring of global and national actors by independent institutions or coalitions, to improve donor accountability in general and to recipient countries in particular [60].

The SWAp experience highlights the importance of continuity, based on stable relationships among the major international actors and with recipient countries, and the ability to rise above the turbulence caused by short term priorities, funding cycles, and transient personal agendas. This requires a sustained focus on long-term objectives [49], eschewing of donor shifts towards 'fashionable' initiatives. However, in many cases this will require a new mindset among those involved and a commitment from 'recipient' governments to maintain their investment in health when donors are making substantial contributions.

Just as at national level, there is a need to boost capacity at international level. As already noted, the body of knowledge that can be drawn upon to guide decision making is limited and there is often inadequate understanding of the extent to which findings are contextually bounded. Many major donors and national governments face difficulties in recruiting individuals with appropriate skills and experience but this problem is especially great in the area of health systems. Translation of research into policy-relevant messages is often weak, with considerable scope to develop and evaluate cadres of knowledge brokers to function as intermediaries between researchers and policymakers [66]. Although communication involves transmission and reception, there has often been inadequate investment in the creation of research aware policy-makers and practitioners in recipient countries. This is, however, being addressed in an important new initiative, EVIPNet, (Evidence-Informed Policy Network For Better Decision Making) [67]. This is a WHO initiative that brings together researchers, policy makers and civil society to facilitate the use of high quality research evidence by policy-makers in low and middle-income countries.

Finally, there is a need to strengthen systems of accountability, at global, national and sub-national level. This will never be easy, given the diversity of bodies involved and the fact that development assistance is intrinsically political. There is also a need for a better understanding of the role of the private and voluntary sectors, the interests they represent, and the ways in which they can be held accountable. Governments and private donors cannot be made to do something they would otherwise not do. However, they are not immune from public opinion, especially when a light is shone on their activities by non-governmental agencies, such as Global Health Watch [68].

## Conclusions

The need for health systems support to recipient countries now appears well accepted, but there is currently no clear way forward as to how this should implemented. Indeed, there is a risk that efforts to address health system strengthening will simply aggravate the current crowded scene of diverse global initiatives. Simply adding health system funding streams to existing global initiatives is not enough. There needs to be radical action to simplify the global health architecture and reduce the transactions costs they impose on countries. Five key actions are needed.

First, there is a need for agreement on which international agencies, or partnerships, should be involved in health systems strengthening and in what ways, taking into account their mandates, expertise, and comparative advantage [69]. In particular, there is a need for clarity on who can provide leadership and on the roles of the WHO and the World Bank. It will also be important to learn lessons from the implementation of the International Health Partnership.

Second, it is vital that recipient countries should have, or should be supported to develop, a coherent national strategy for prioritizing external support and managing it together with local resources in a coordinated way. There should be effective agreements that standardize health worker remuneration, so that one initiative does not poach staff from another, although reaching such agreements may be difficult as they must take account of local labor market conditions and, especially, the need for some form of equivalence with the private sector. Parallel systems should not be created, whether to procure drugs or account for expenditures. Given the scarcity of resources for delivery of care, agencies that provide disease specific funding should be required to ensure they do not free-ride on a health system infrastructure which they do not support.

Third, while many countries require greater technical capacity in managing and controlling specific diseases, and national or regional disease control programs have considerable value, it is vital that disease control activities are integrated with other health services at the level of service delivery. In particular, there are substantial synergies in delivery of care to those with AIDS and non-communicable diseases, both of which require well-managed pharmaceutical supply chains, trained multi-professional teams, and management systems to support long-term relationships between patients and health care providers at different levels of the system. The exceptions would be where the nature of the intervention is such that it is free-standing - as with national media campaigns, for example, or interventions in other sectors such as schools.

Fourth, advocates of health system support need to give more careful thought to how the success of health systems strengthening can be assessed. Historically, health systems have been seen as not just a black box but also a black hole, absorbing resources without visible results [51]. The challenge is to hold health systems accountable for performance, but without resorting to narrow targets that can distort behavior, as has been the experience in the UK NHS, for example [70]. It is easy to see how the unsophisticated use of targets might encourage an organization to concentrate its efforts in urban areas where populations are most accessible, to the detriment of those in remote settings [71] or, as described with the GFATM's activities in Nicaragua, might create pressure to sacrifice quality for quantity [72].

Finally, the evidence base from which countries can draw examples of successful approaches to improving health systems performance is extremely weak. There is an urgent need for greater investment in applied health systems research in low income countries, especially that focusing on implementation of effective approaches to improving health system performance, including training of researchers and strengthening of research institutions [73]. This investment should recognize the importance of research and evaluation that draws on a wide range of disciplinary perspectives and uses both quantitative and qualitative methods. This should increase the evidence base for system interventions as well as develop methods for monitoring progress. Such research should take account of the effect of context on implementation, where possible embedding analysis in a systems framework that recognizes the existence of complexity, characterized by dynamic relationships, variable time lags and feedback loops [74,75]. Approaches which help translate evidence into policy, such as knowledge broking initiatives like EVIPNet must also be supported.

Successful implementation of these five steps should increase the prospects substantially for attainment of the MDGs, as well as enabling health systems to respond to other health challenges.

## Competing interests

The authors declare that they have no competing interests.

## Authors' contributions

DB and MM jointly drafted the manuscript. AM, GW, and AH revised it. All authors read and approved the final manuscript.

## References

[B1] Organisation for Eeconomic Co-operation and Development Aid effectiveness in health 2006 Paris: OECD

[B2] Reich MR Takemi K Roberts MJ Hsiao WC Global action on health systems: a proposal for the Toyako G8 summit Lancet 2008 371 865 869 10.1016/S0140-6736(08)60384-0 18328932

[B3] Subramanian S Peters D Willis J How are Health Services, Financing and Status Evaluated? An Analysis of Implementation Completion Reports of World Bank Assistance in Health 2006 Washington DC: World Bank

[B4] Haines A Contribution of health systems to disease control Trop Med Int Health 2007 12 1275 1278 18045259 10.1111/j.1365-3156.2007.01934.x

[B5] Victora CG Huicho L Amaral JJ Armstrong-Schellenberg J Manzi F Mason E Scherpbier R Are health interventions implemented where they are most needed? District uptake of the integrated management of childhood illness strategy in Brazil, Peru and the United Republic of Tanzania Bull World Health Organ 2006 84 792 801 10.2471/BLT.06.030502 17128359 PMC2627500

[B6] Bloom G Standing H Future health systems: Why future? Why now? Social Science and Medicine 2008 66 2067 2075 10.1016/j.socscimed.2008.01.032 18321628

[B7] Frenk J The global health system: strengthening national health systems as the next step for global progress PLoS Med 2010 7 e1000089 10.1371/journal.pmed.1000089 20069038 PMC2797599

[B8] Mills A Rasheed F Tollman S Jamison DT, Breman JG, Measham AR, Alleyne G, Claeson M, Evans DB, Jha P, Mills A, Musgrove P Strengthening Health Systems Disease Control Priorities in Developing Countries 2006 2 Oxford: Oxford University Press 87 102 21250325

[B9] Samb B Evans T Dybul M Atun R Moatti JP Nishtar S Wright A Celletti F Hsu J Kim JY An assessment of interactions between global health initiatives and country health systems Lancet 2009 373 2137 2169 10.1016/S0140-6736(09)60919-3 19541040

[B10] WHO The World Health Report 2000. Health Systems: Improving Performance 2000 Geneva: World Health Organisation

[B11] Marchal B Cavalli A Kegels G Global health actors claim to support health system strengthening - is this reality or rhetoric? PLoS Med 2009 6 e1000059 doi:1000010. 1001371/journal.pmed. 1000059 10.1371/journal.pmed.1000059 19399158 PMC2667637

[B12] Shiffman J Has donor prioritization of HIV/AIDS displaced aid for other health issues? Health Policy Plan 2008 23 95 100 10.1093/heapol/czm045 18156161

[B13] Acharya A De Lima A Moore M Proliferation and Fragmentation: Transactions Costs and the Value of Aid Journal of Development Studies 2006 42 1 21 10.1080/00220380500356225

[B14] Ooms G Van Damme W Baker BK Zeitz P Schrecker T The 'diagonal' approach to Global Fund financing: a cure for the broader malaise of health systems? Global Health 2008 4 6 10.1186/1744-8603-4-6 18364048 PMC2335098

[B15] Lu C Schneider MT Gubbins P Leach-Kemon K Jamison D Murray CJL Public financing of health in developing countries: a cross-national systematic analysis Lancet 2010 375 1375 1387 10.1016/S0140-6736(10)60233-4 20381856

[B16] Stuckler D Basu S McKee M What causes aid displacement? International Monetary Fund lending programmes reduce health system spending International Journal of Health Services in press 10.2190/HS.41.1.e 21319721

[B17] Brugha R Donoghue M Starling M Ndubani P Ssengooba F Fernandes B Walt G The Global Fund: managing great expectations Lancet 2004 364 95 100 10.1016/S0140-6736(04)16595-1 15234862

[B18] Hard Currency. Unilateralism doesn't work for foreign aid either http://www.washingtonmonthly.com/features/2004/0403.birdsall.html

[B19] Xu K Evans DB Carrin G Aguilar-Rivera AM Musgrove P Evans T Protecting households from catastrophic health spending Health Aff (Millwood) 2007 26 972 983 10.1377/hlthaff.26.4.972 17630440

[B20] China's Money Flows West http://www.newsweek.com/2009/03/13/china-s-money-flows-west.html#

[B21] Adeyi O Smith O Robles S Public Policy and the Challenge of Chronic Noncommunicable Diseases 2007 Washington DC: World Bank

[B22] UNAIDS Financial Resources Required to Achieve Universal Access to HIV Prevention, Treatment, Care and upport 2007 Geneva: UNAIDS

[B23] Beran D Yudkin JS de Courten M Access to care for patients with insulin-requiring diabetes in developing countries: case studies of Mozambique and Zambia Diabetes Care 2005 28 2136 2140 10.2337/diacare.28.9.2136 16123479

[B24] Beaglehole R Epping-Jordan J Ebrahim S Chopra M Patel V Kidd M Haines A Improving the management of chronic disease in low- and middle- income countries: a priority for primary health care Lancet 2008 372 940 949 10.1016/S0140-6736(08)61404-X 18790317

[B25] Atun R de Jongh T Secci FV Ohiri K Adeyi O Clearing the global health fog: a systematic review of the evidence on integration of targeted health interventions into health systems and targeted interventions 2009 Washington, DC: World Bank

[B26] Walton DA Farmer PE Lambert W Leandre F Koenig SP Mukherjee JS Integrated HIV prevention and care strengthens primary health care: lessons from rural Haiti J Public Health Policy 2004 25 137 158 10.1057/palgrave.jphp.3190013 15255381

[B27] Price JE Leslie JA Welsh M Binagwaho A Integrating HIV clinical services into primary health care in Rwanda: a measure of quantitative effects AIDS Care 2009 21 608 614 10.1080/09540120802310957 19444669

[B28] World Health Organization Community-directed interventions for major health problems in Africa: a multi-country study: final report. WHO, special programme for Research and Training in Tropical Diseases 2008 Geneva: WHO

[B29] World Health Organization The World Health Report 2006 - Working together for health 2006 Geneva: WHO

[B30] Dubois CA McKee M Nolte E Human resources for health in Europe 2005 Buckingham: Open University Press

[B31] Rese A Balabanova D Danishevski K McKee M Sheaff R Implementing general practice in Russia: getting beyond the first steps British Medical Journal 2005 331 204 207 10.1136/bmj.331.7510.204 16037457 PMC1179768

[B32] Mendis S Fukino K Cameron A Laing R Filipe A Jr Khatib O Leowski J Ewen M The availability and affordability of selected essential medicines for chronic diseases in six low- and middle-income countries Bull World Health Organ 2007 85 279 288 10.2471/BLT.06.033647 17546309 PMC2636320

[B33] The Bamako call to action on research for health. Strengthening research for health, development, and equity http://apps.who.int/gb/ebwha/pdf_files/EB124/B124_12Add2-en.pdf

[B34] McKee M Global research for health British Medical Journal 2008 337 1249 1250 10.1136/bmj.a1249

[B35] Saltman RB Ferroussier-Davis O The concept of stewardship in health policy Bull World Health Organ 2000 78 732 739 10916910 PMC2560782

[B36] Newbrander W Rebuilding Health Systems and Providing Health Services in Fragile States 2007 Cambridge MA: Management Sciences for Health

[B37] Vian T Review of corruption in the health sector: theory, methods and interventions Health Policy Plan 2008 23 83 94 10.1093/heapol/czm048 18281310

[B38] Nolte E McKee M Caring for people with chronic conditions. A health system perspective 2008 Maidenhead: Open University Press

[B39] Daar AS Singer PA Persad Deepa Leah Pramming Stig K Matthews David R Beaglehole Robert Bernstein Alan Borysiewicz Leszek K Colagiuri Stephen Ganguly Nirmal Grand challenges in chronic non-communicable diseases. The top 20 policy and research priorities for conditions such as diabetes, stroke and heart disease. Poor diet and smoking are two factors that contribute to the millions of preventable deaths that occur each year Nature 2007 450 494 496 10.1038/450494a 18033288

[B40] Ensor T Cooper S Overcoming barriers to health service access: influencing the demand side Health Policy Plan 2004 19 69 79 10.1093/heapol/czh009 14982885

[B41] Cohen J Global health. The new world of global health Science 2006 311 162 167 10.1126/science.311.5758.162 16410496

[B42] Schieber GJ Gottret P Fleisher LK Leive AA Financing global health: mission unaccomplished Health Aff (Millwood) 2007 26 921 934 10.1377/hlthaff.26.4.921 17630434

[B43] Balogun P Evaluating Progress Towards Harmonisation. Working paper 15 2005 London: DfID

[B44] Biesma RG Brugha R Harmer A Walsh A Spicer N Walt G The effects of global health initiatives on country health systems: a review of the evidence from HIV/AIDS control Health Policy Plan 2009 24 239 252 10.1093/heapol/czp025 19491291 PMC2699244

[B45] Taskforce on Innovative International Financing for Health Systems More money for health, and more health for the money ...to achieve the health MDGs, ...to save the lives of millions of women and children, and ...to help babies in low-income settings have a safer start to life 2009 London: Taskforce on Innovative International Financing for Health Systems

[B46] WHO The Global Fund's Strategic Approach To Health System Strengthening Consultation 2007 vol. Background note 4

[B47] World Bank Healthy Development. The World Bank Strategy for Health, Nutrition, and Population Results 2007 Washington DC: World Bank

[B48] McCoy D The World Bank's new health strategy: reason for alarm? Lancet 2007 369 1499 1501 10.1016/S0140-6736(07)60688-6 17482968

[B49] Department for International Development Working together for better health 2007 London: DfID

[B50] International Labour Office An ILO strategy towards universal access to health care [Draft for consultation] 2007 Geneva: International Labour Office

[B51] Frenk J Strengthening Health Systems: Towards New Forms of Global Cooperation Meeting on Global Health and the United Nations 2008 Carter Centre, Atlanta, Georgia, USA

[B52] World Health Organization Maximizing positive synergies between health systems and Global Health Initiatives. Report on the expert consultation on positive synergies between health systems and GHIs 2008 Geneva: WHO

[B53] Sepúlveda J Jamison DT, Breman JG, Measham A, Alleyne G, Claeson M, Evans DB, Jha P, Mills A, Musgrove P Foreword Disease control priorities in developing countries 2006 2 Washington, DC: Oxford University Press 21250309

[B54] World Health Organization The Global Fund's Strategic Approach to Health Systems Strengthening. Report from WHO to the Global Fund Secretariat 2007 Geneva: WHO

[B55] Frenk J Bridging the divide: global lessons from evidence-based health policy in Mexico Lancet 2006 368 954 961 10.1016/S0140-6736(06)69376-8 16962886

[B56] Lewin S Lavis JN Oxman AD Bastias G Chopra M Ciapponi A Flottorp S Marti SG Pantoja T Rada G Supporting the delivery of cost-effective interventions in primary health-care systems in low-income and middle-income countries: an overview of systematic reviews Lancet 2008 372 928 939 10.1016/S0140-6736(08)61403-8 18790316

[B57] Mills A Bennett S Russell S Attanayake N Hongoro C Muraleedharan VE Smithson P The challenge of health sector reform: what must governments do? 2001 Oxford: Macmillan Press

[B58] Chan M Kazatchkine M Lob-Levyt J Obaid T Schweizer J Sidibe M Veneman A Yamada T Meeting the Demand for Results and Accountability: A Call for Action on Health Data from Eight Global Health Agencies PLoS Med 2010 7 e1000223 doi:1000210. 1001371/journal.pmed. 1000223 10.1371/journal.pmed.1000223 20126260 PMC2811154

[B59] Global Fund to fight AIDS TB and Malaria Health Systems Strenghthening7th Policy and Strategy Committee Meeting 2007 Geneva: GFATM

[B60] Global Economic Governance Programme Setting a Developing Country Agenda for Global Health. Preliminary Report of a High-Level Working Group 2008 Centre for International Studies, University College Oxford

[B61] OECD Aid effectiveness in health 2006 Paris: OECD

[B62] WHO Maximizing positive synergies between health systems and Global Health Initiatives. Report on the expert consultation on positive synergies between health systems and GHIs 2008 Geneva

[B63] Tashobya C Ssengooba F Oliveira-Cruz V Health systems reforms in Uganda: processes and outputs 2006 London: LSHTM, Health Systems Development Programme

[B64] Sundewall J Sahlin-Andersson K Translations of health sector SWAps--a comparative study of health sector development cooperation in Uganda, Zambia and Bangladesh Health Policy 2006 76 277 287 10.1016/j.healthpol.2005.06.009 16039002

[B65] Greco G Powell-Jackson T Borghi J Mills A Countdown to 2015: assessment of donor assistance to maternal, newborn, and child health between 2003 and 2006 Lancet 2008 371 1268 1275 10.1016/S0140-6736(08)60561-9 18406861

[B66] Green A Bennett S Sound Choices. Enhancing Capacity for Evidence-Informed Health Policy 2007 Geneva: Alliance for Health Policy and Systems Research. WHO

[B67] Corkum S Cuervo LG Porras A EVIPNet Americas: informing policies with evidence Lancet 2008 372 1130 1131 10.1016/S0140-6736(08)61459-2 18926261

[B68] Global Health Watch Global Health Watch 2005-6 2007 Cape Town: Global Health Watch

[B69] Szlezak NA Bloom BR Jamison DT Keusch GT Michaud CM Moon S Clark WC The global health system: actors, norms, and expectations in transition PLoS Med 2010 7 e1000183 10.1371/journal.pmed.1000183 20052277 PMC2796301

[B70] Wismar M McKee M Busse R E K Targets for health: uses and abuses 2008 Brussels: European Observatory on Health Systems and Policies

[B71] Hanefeld J How have Global Health Initiatives impacted on health equity? Promot Educ 2008 15 19 23 10.1177/1025382307088094 18430691

[B72] Plamondon KM Hanson L Labonte R Abonyi S The Global Fund and tuberculosis in Nicaragua: building sustainable capacity? Can J Public Health 2008 99 355 358 18767286 10.1007/BF03403771 PMC6975681

[B73] Sanders D Haines A Implementation research is needed to achieve international health goals PLoS Med 2006 3 e186 10.1371/journal.pmed.0030186 16729844 PMC1472551

[B74] Atun R Menabde N Coker R, Atun R, McKee M Health systems and systems thinking Health Systems and the Challenge of Communicable Disease Experiences from Europe and Latin America 2005 Buckingham: Open University Press 121 140

[B75] de Savigny D Adam T Systems thinking for health systems strengthening 2009 Geneva: Alliance for Health Policy and Systems Research/WHO

